# Supplementary material for: A mechanical transition from tension to buckling underlies the jigsaw puzzle shape morphogenesis of histoblasts in the Drosophila epidermis
Source: PLoS Biol. 2024 Jun 13;22(6):e3002662. doi: 10.1371/journal.pbio.3002662 (PMC11175506; doi:10.1371/journal.pbio.3002662)
Supplement: S1 Text — Table A. Statistical tests for pair-wise comparison of the distributions presented in Fig 1. Table B. Normality test for parameter distributions in Fig 1. Table C. Statistical tests of the results presented in Fig 3. Tables D and E. Statistical tests relative to the results shown in Fig 7. Table F. Statistical tests relative to the results shown in S7 Fig. H and p-values reported were calculated by Mann–Whitney U test. (PDF) [file pbio.3002662.s001.pdf]

S1 text : Supplementary Tables

Table A: Statistical tests for pair-wise comparison of the distributions presented in Fig 1.
Table B: Normality test for parameter distributions in Fig 1.
Table C: Statistical tests of the results presented in Fig 3.
Table D,E: Statistical tests relative to the results shown in Fig 7.
Table F: Statistical tests relative to the results shown in Fig S7. H and *p*-values reported were calculated by Mann-Whitney U test.

Table A. Statistical tests for pair-wise comparison of the distributions presented in Fig 1. The table reports the H and *p* values for a Mann-Whitney U test at 0.05 confidence. Samples are numbered according to their age, in h AEL (hours after egg laying). In red, are the conditions for wich the *p*-value is higher than the standard 0.05. wp = white pupa, perim = perimeter, circ = circularity

Mann-Whitney U test - H

	Area - < 90h	Area - 90-95h	Area - 95-100h	Area - >100h	Area - wp
**Area - < 90h**	0				
**Area - 90-95h**	0	0			
**Area - 95-100h**	1	0	0		
**Area - > 100h**	1	1	1	0	
**Area - wp**	1	1	1	1	0

Mann-Whitney U test - p

	Area - < 90h	Area - 90-95h	Area - 95-100h	Area - >100h	Area - wp
**Area - < 90h**	1				
**Area - 90-95h**	4,47E-01	1			
**Area - 95-100h**	2,68E-02	6,85E-02	1		
**Area - > 100h**	3,95E-24	2,02E-30	7,02E-20	1	
**Area - wp**	5,16E-24	2,97E-27	1,49E-21	1,68E-07	1

Mann-Whitney U test - H

	Perim - < 90h	Perim - 90-95h	Perim - 95-100h	Perim - >100h	Perim - wp
**Perim - < 90h**	0				
**Perim - 90-95h**	1	0			
**Perim - 95-100h**	1	0	0		
**Perim - > 100h**	1	1	1	0	
**Perim - wp**	1	1	1	1	0

Mann-Whitney U test - p

	Perim - < 90h	Perim - 90-95h	Perim - 95-100h	Perim - >100h	Perim - wp
**Perim - < 90h**	1				
**Perim - 90-95h**	1,43E-04	1			
**Perim - 95-100h**	3,92E-04	8,53E-01	1		
**Perim - > 100h**	8,59E-30	7,08E-29	2,07E-27	1	
**Perim - wp**	1,51E-07	1,63E-04	2,69E-04	1,43E-05	1

Mann-Whitney U test - H

	Circ - < 90h	Circ - 90-95h	Circ - 95-100h	Circ - >100h	Circ - wp
**Circ - < 90h**	0				
**Circ - 90-95h**	1	0			
**Circ - 95-100h**	1	1	0		
**Circ - > 100h**	1	1	1	0	
**Circ - wp**	1	1	1	1	0

Mann-Whitney U test - p

	Circ - < 90h	Circ - 90-95h	Circ - 95-100h	Circ - >100h	Circ - wp
**Circ - < 90h**	1				
**Circ - 90-95h**	6,76E-12	1			
**Circ - 95-100h**	6,53E-21	1,36E-03	1		
**Circ - > 100h**	3,06E-65	1,97E-68	5,96E-65	1	
**Circ - wp**	2,89E-31	4,13E-31	2,13E-30	1,54E-02	1

Table B. Normality test for parameter distributions in Fig 1. Samples are numbered according to their age, in h AEL (hours after egg laying). wp = white pupa, perim = perimeter, circ = circularity

	Lilliefors test		Anderson-Darling test	
	**H**	***p***	**H**	***p***
**Area - $<$ 90h**	0	1,91E-01	1	1,73E-02
**Area - 90-95h**	1	1,15E-02	1	4,35E-03
**Area - 95-100h**	1	1,00E-03	1	5,00E-04
**Area - $>$ 100h**	1	1,00E-03	1	5,00E-04
**Area - wp**	1	3,96E-02	1	1,65E-03
**Perim- $<$ 90h**	0	1,66E-01	0	7,95E-02
**Perim- 90-95h**	1	3,88E-02	0	2,16E-01
**Perim- 95-100h**	1	1,00E-03	1	5,00E-04
**Perim- $>$ 100h**	1	1,00E-03	1	5,00E-04
**Perim- wp**	0	6,87E-02	1	3,85E-03
**Circ - $<$ 90h**	0	5,00E-01	0	7,02E-01
**Circ - 90-95h**	0	1,60E-01	0	9,65E-02
**Circ - 95-100h**	1	1,00E-03	1	5,00E-04
**Circ - $>$ 100h**	1	1,00E-03	1	5,00E-04
**Circ - wp**	1	1,00E-03	1	5,00E-04

Table C. Statistical tests of the results presented in Fig 3. H and *p*-values reported were calculated by Mann-Whitney U test. wt: wild type; wwrt: with respect to;

	median	Area - H wrt wt	Area - p wrt wt
**wt**	135,30		
**Rab11**	50,12	1	1,67E-26
**Stg**	20,06	1	1,11E-137
**Cad-RNAi**	138.22	0	1,94E-1

	median	Perim - H wrt wt	Perim - p wrt wt
**wt**	99,69		
**Rab11**	30,01	1	3,80E-32
**Stg**	18,56	1	2,14E-143
**Cad-RNAi**	52,43	1	7,3E-19

	median	Circ - H wrt wt	Circ - p wrt wt
**wt**	0,17		
**Rab11**	0,74	1	4,01E-38
**Stg**	0,75	1	4,57E-145
**Cad-RNAi**	0,62	1	5,2E-25

Table D. Statistical tests relative to the results shown in Fig 7. H and *p*-values reported were calculated by Mann-Whitney U test. wt: wild type; wrt: with respect to

	median	Area - H wrt wt	Area - p wrt wt
**wt**	135,30		
**InR-DN**	181,88	1	2,48E-02
**TSC1-RNAi**	87,51	1	3,60E-08

Table E. Statistical tests corresponding to the data shown in Fig 7D. H and *p*-values reported were calculated by Mann-Whitney U test.

	WT	InRdn	TSC1
**Area (µm2)**	1.5004E+03	1.0042 E+03	1.8567E+03
**p (Mann-Whitney) vs WT**		0,0142	0,0228

Table F. Statistical tests relative to the results shown in Fig S7. H and *p*-values reported were calculated by Mann-Whitney U test.

	median		wt_wp VS InRdn_wp	
	**wt**	**InRdn**	**H**	**p**
**area**	88,29	155,71	1	1,32E-07
**circ**	0,22	2,78E-01	1	9,77E-04
