# Supplementary figures and images for: A mechanical transition from tension to buckling underlies the jigsaw puzzle shape morphogenesis of histoblasts in the Drosophila epidermis
Source: PLoS Biol. 2024 Jun 13;22(6):e3002662. doi: 10.1371/journal.pbio.3002662 (PMC11175506; doi:10.1371/journal.pbio.3002662)

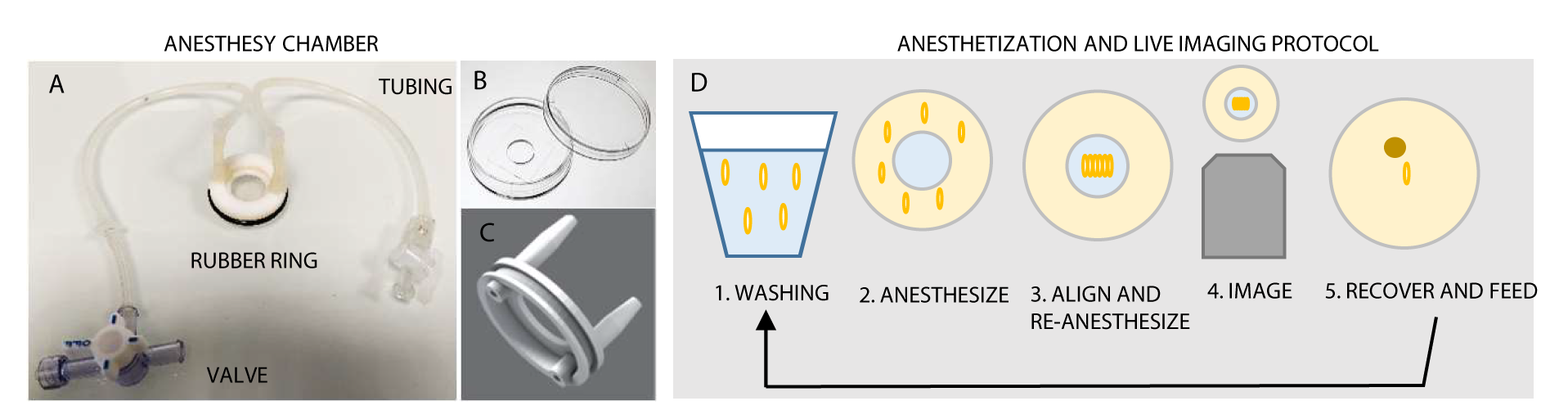

Supplement: S1 Fig — (A) Assembled anesthesia chamber made of a glass-bottom dish (zoom in B), a custom-designed injection lid (zoom in C), tubing, and valves to which syringes are then connected. (D) Main steps of the imaging protocol: (1) larvae are washed in PBS and dried on a lab wipe; (2) larvae are positioned around the glass of the Petri dish and anesthetized for 5 min; (3) after closing the valves to keep the anesthetic, larvae are aligned on the glass, with a drop of halo-carbon oil; 4) larvae are images by confocal spinning disc microscopy; and (5) for chronic imaging, each larva is placed in a Petri dish with soft food and let recover for a few hours before repeating the procedure. (TIF) [file pbio.3002662.s002.tif]

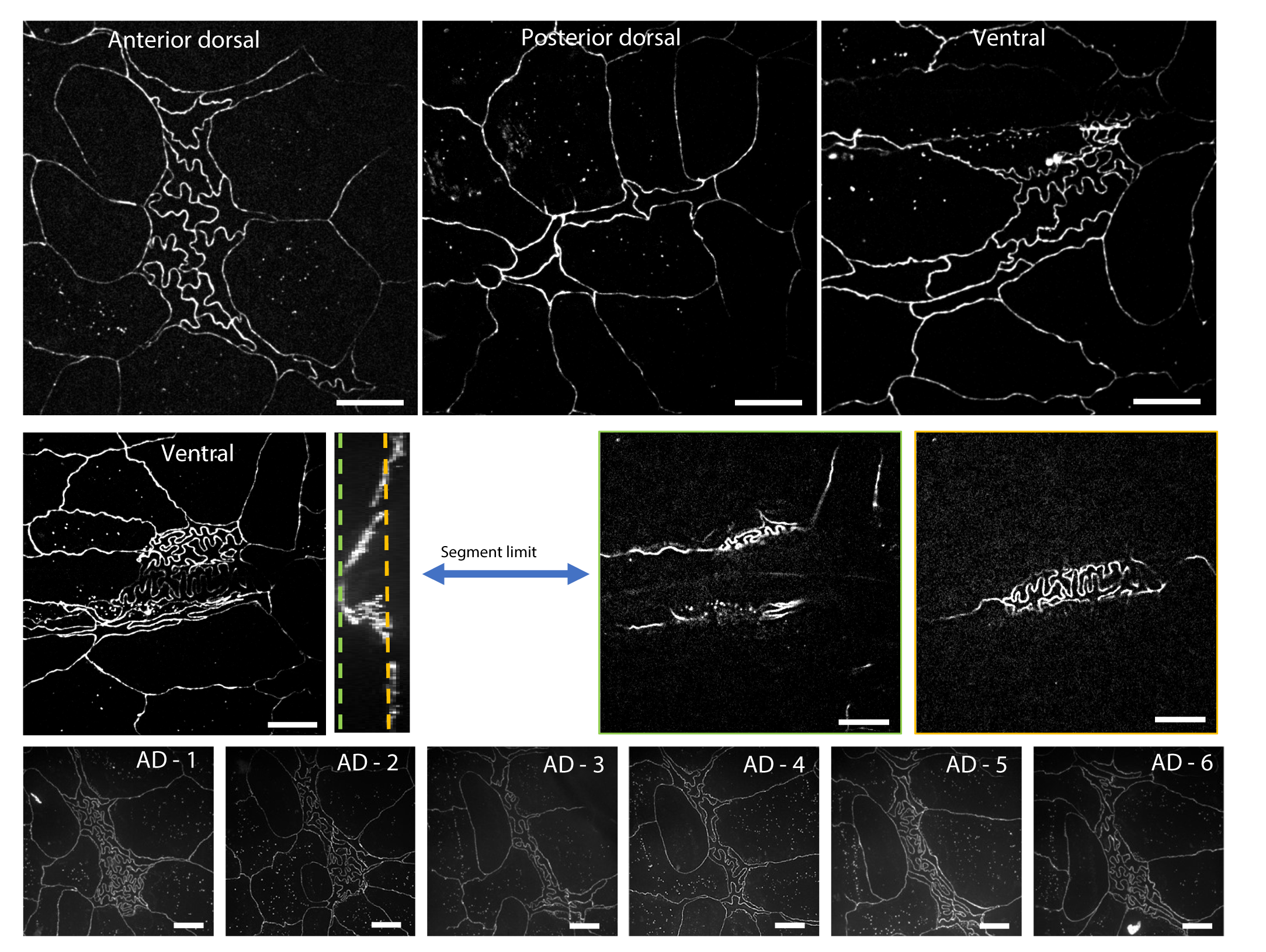

Supplement: S2 Fig — Upper panel: anterior dorsal, posterior dorsal, and ventral histoblast nests from the sixth segment of the same larva at the wandering stage are shown. Histoblasts from all nests show the same buckling phenotype. We chose to image the anterior dorsal nests because of 2 main reasons: they are formed by a higher cell number, and they are flatter than the ventral nests, which are often located close to the limit between 2 abdominal segments. Middle panel: abdominal nest from the same larva, which features a deep fold due to the location close to the segment limit. This makes the image quality low, and the analysis less reliable. Lower panel: anterior dorsal nests from 1 to 6 of the same wandering larva. Scale bars = 20 μm. (TIF) [file pbio.3002662.s003.tif]

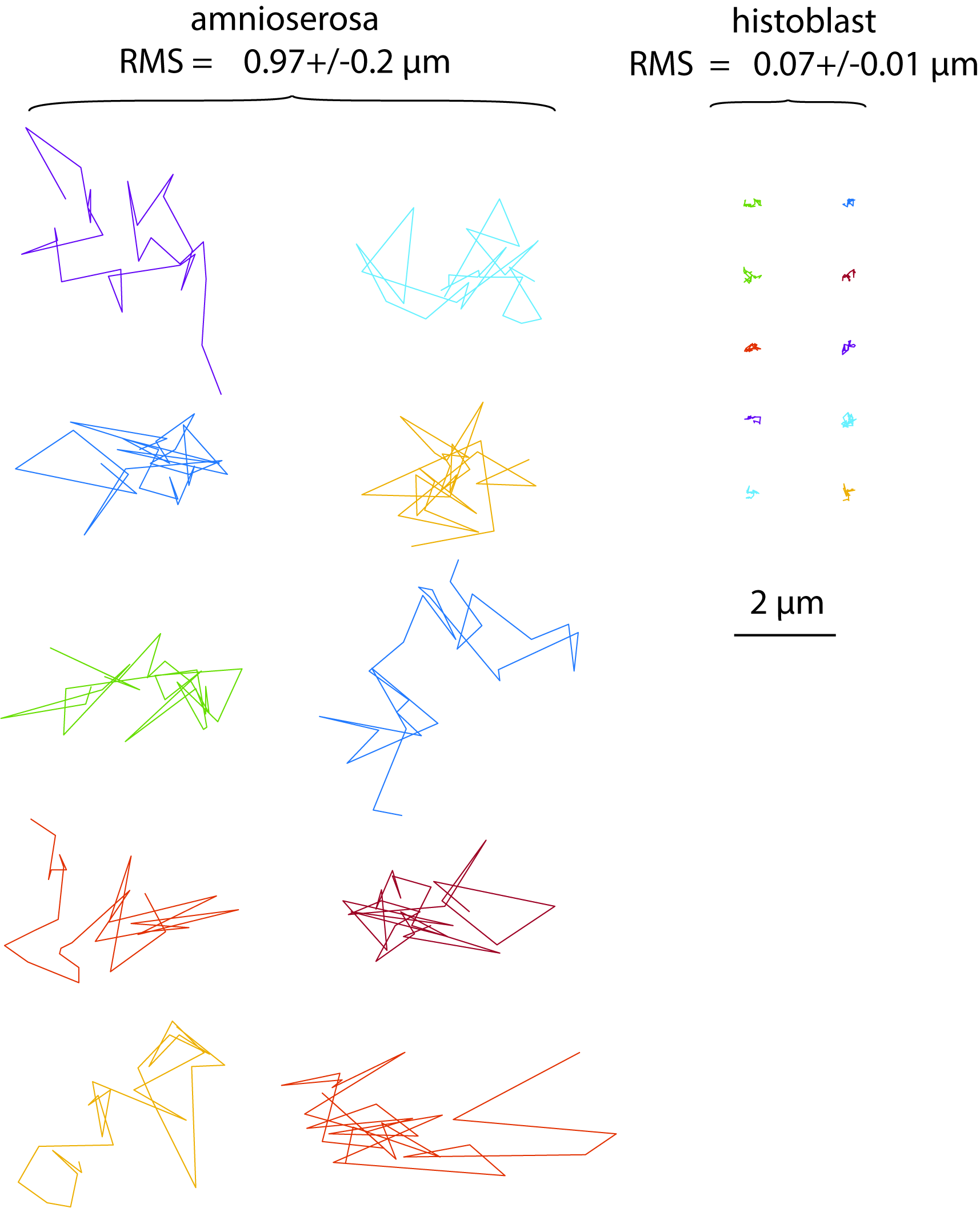

Supplement: S3 Fig — Ten trajectories of vertices in the amnioserosa at the onset of dorsal closure are show on the left. Ten trajectories of vertices of histoblast from wandering stage larva are shown on the right. Thirty points were measured with a time interval of 1 min. In vertices from the amnioserosa, a global drift due to morphogenetic movement was removed by subtracting the displacement of the center of mass of all vertices. (TIF) [file pbio.3002662.s004.tif]

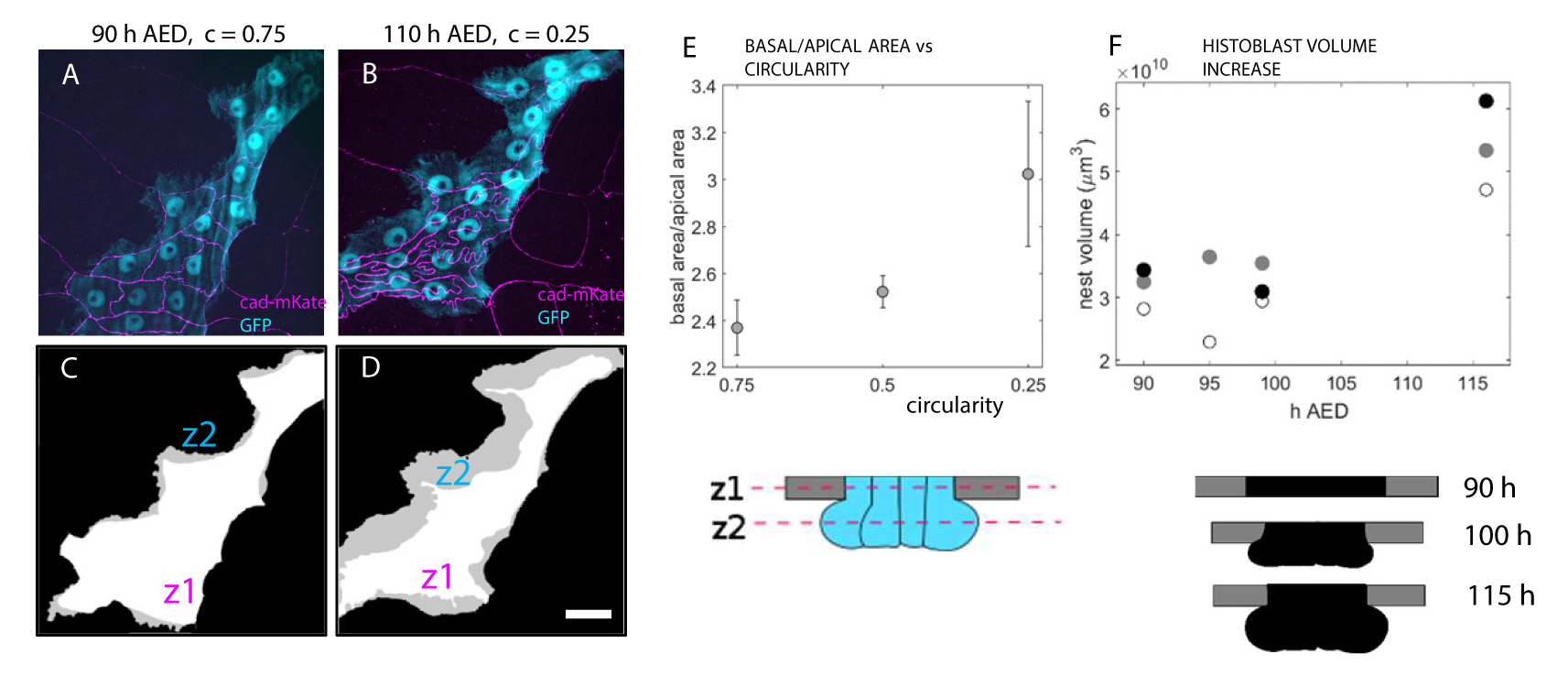

Supplement: S4 Fig — (A) Z-projection of cad:mKate (magenta) and cytosolic GFP (cyan) taken in a larva at 90 h AEL, i.e., at the beginning of the buckling transition. (B) Z-projections of the same larva 20 h later, after the formation of junctional lobules. (C, D) Masks of the external contour of the apical and basal sides of the larva in (A). The white area is obtained from the apical plane obtained from cad:mkate (z1 in the schematic representation), the gray area from the cytosolic GFP maximum projections. It corresponds to the basal plane at which the nest is the largest (z2 in the schematic representations). Before the transition (C) z1 and z2 are almost superposed, while after transition (D) z2 is much bigger than z1, meaning that HBs expand basally. (E) Ratio of basal/apical area for different circularity values, obtained from the masks of the z-projections as shown in C and D, i.e., z2/z1. As HBs junctions fold, the basal areal becomes larger than the apical. Apical and basal areas correspond to the the adherens region and the largest basal area, as schematized below the plot. (F) Total nest volume at different times. White, gray, and black dots correspond each to one histoblasts nest. As apical surface shrink, histoblasts expand below the adherens region, as schematized below the plot. The data underlying the graphs shown in the figure can be found in S1 Data. (TIF) [file pbio.3002662.s005.tif]

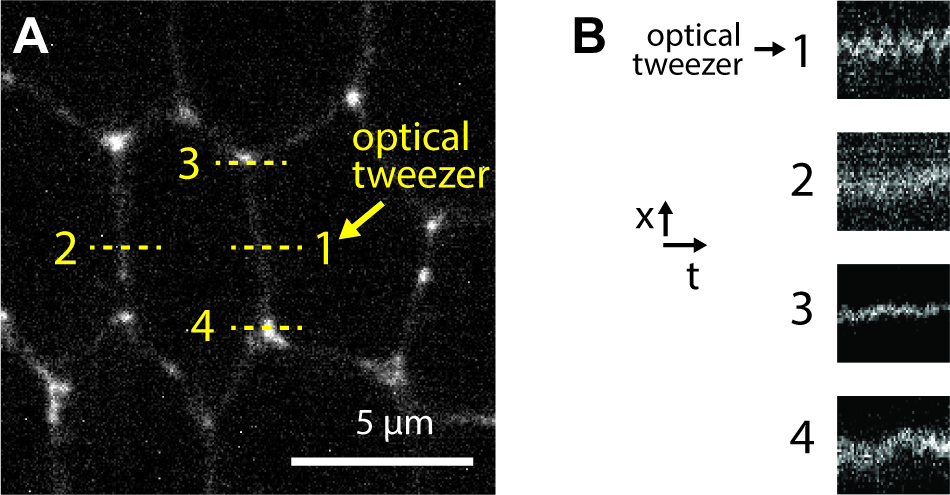

Supplement: S5 Fig — (A) An optical tweezer is used to oscillate a junction (point 1 on the image) in the germ band at the onset of germ band extension. (B) Kymographs display an absence of oscillations at different points around the stimulation. (TIF) [file pbio.3002662.s006.tif]

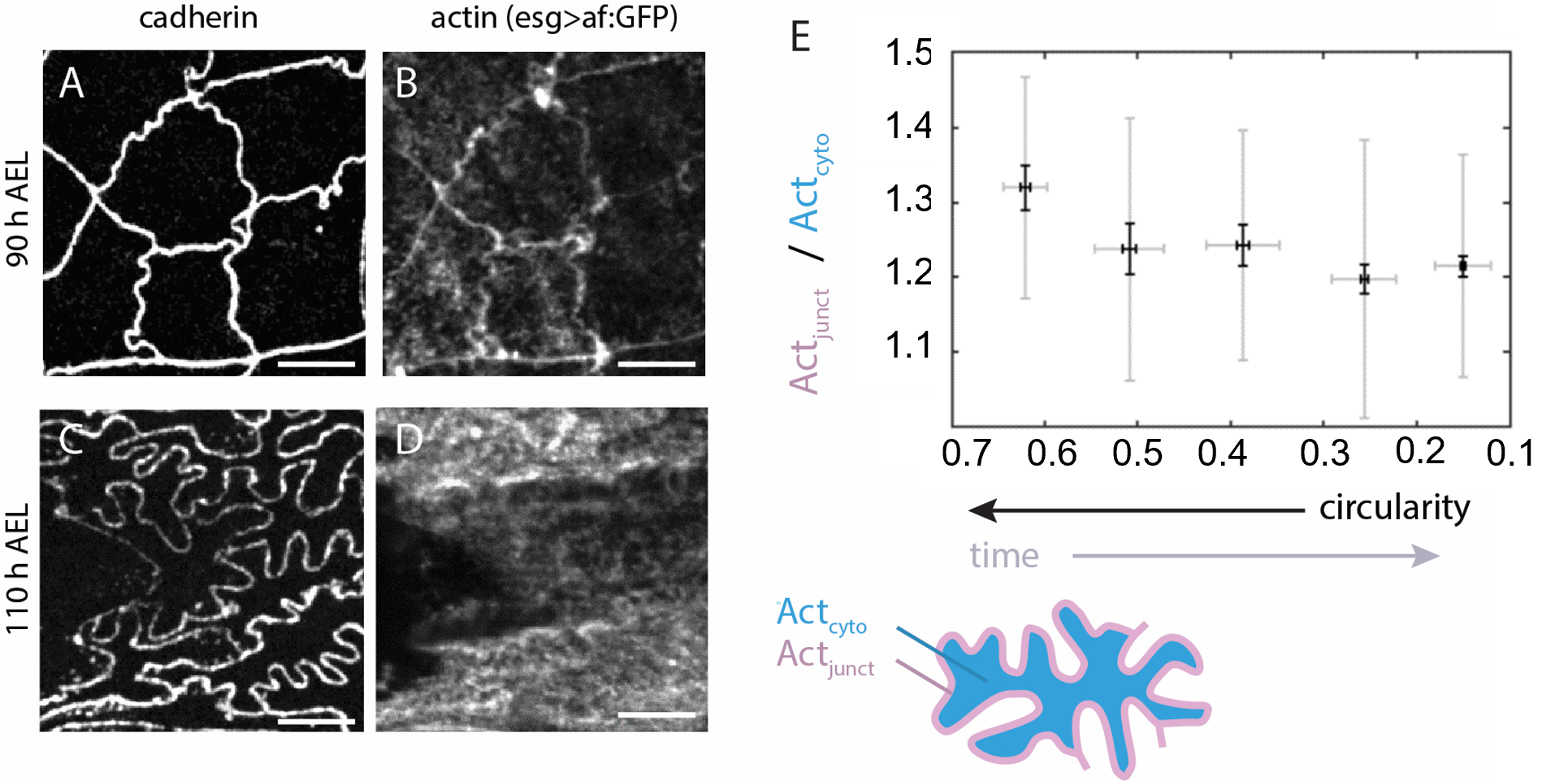

Supplement: S6 Fig — (A-D) Live imaging of cadherin and actin at the level of junctions at 90 h AEL (A, B) and 110 h AEL (C, D). ZX and ZY orthogonal views in (D) signal show the actin enrichment at the tricellular interfaces along the apico-basal axis (arrows). Scale bar = 10 μm. (E) Plot of relative amount of junctional actin as a function of circularity, calculated as the ratio junctional signal Actjunct over cytosolic signal Actcyto as represented in the schematic. Note that the axis of circularity has been inverted to reflect temporality. In the box plot, the horizontal bar represents the median for each bean, the shadowed areas the confidence interval of 0.05, the diamonds correspond to the mean value for each bin and the yellow circles are single data points. Pearson’s correlation coefficients calculated on all the data where 0.18 with a p-value of 0.002. t Test comparisons for the junctional enrichment of the first and last point gave p-values of 0.002. For each bin, N = 116, 88, 32, 27, 24. The data underlying the graphs shown in the figure can be found in S1 Data. (TIF) [file pbio.3002662.s007.tif]

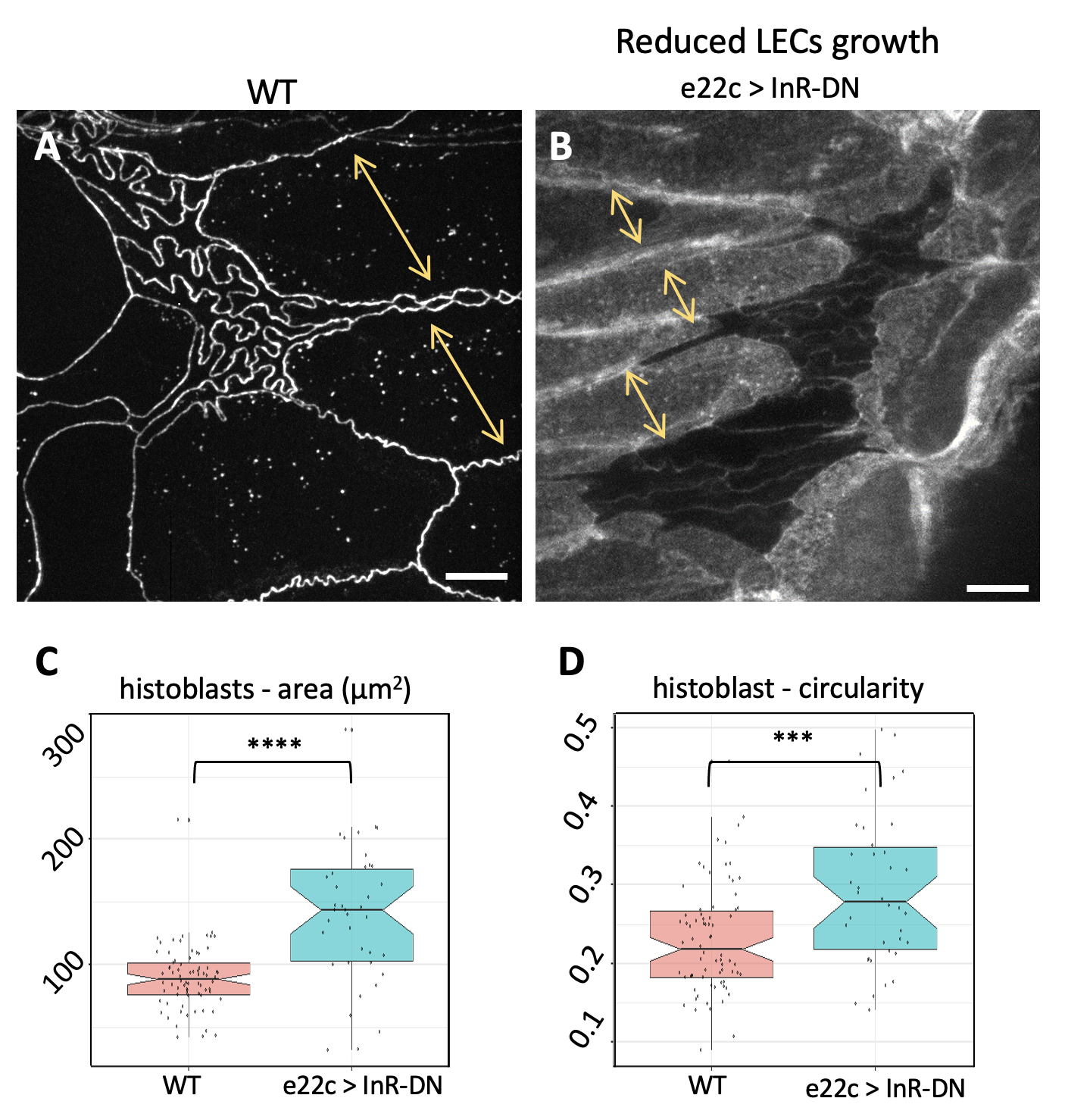

Supplement: S7 Fig — (A) Live imaging of the epidermis of a WT white pupa. (B) Live imaging of the epidermis of a white pupa in which LECs growth was reduced by impairing the insulin receptor pathway, causing reduced load on histoblast junctions, hence reduced junctional buckling as compared to the WT. Yellow arrows in both indicate the width of LECs, which increases in TSC1-RNAi and decreases in InR-DN larvae. Scale bar = 10 μm. (C, D) Box plots showing the quantifications of histoblasts area in WT and InRdn white pupae. p-Values were calculated by Mann–Whitney U test. N = 74 (WT), N = 38 (InRdn) cells. The data underlying the graphs shown in the figure can be found in S1 Data. (TIF) [file pbio.3002662.s008.tif]

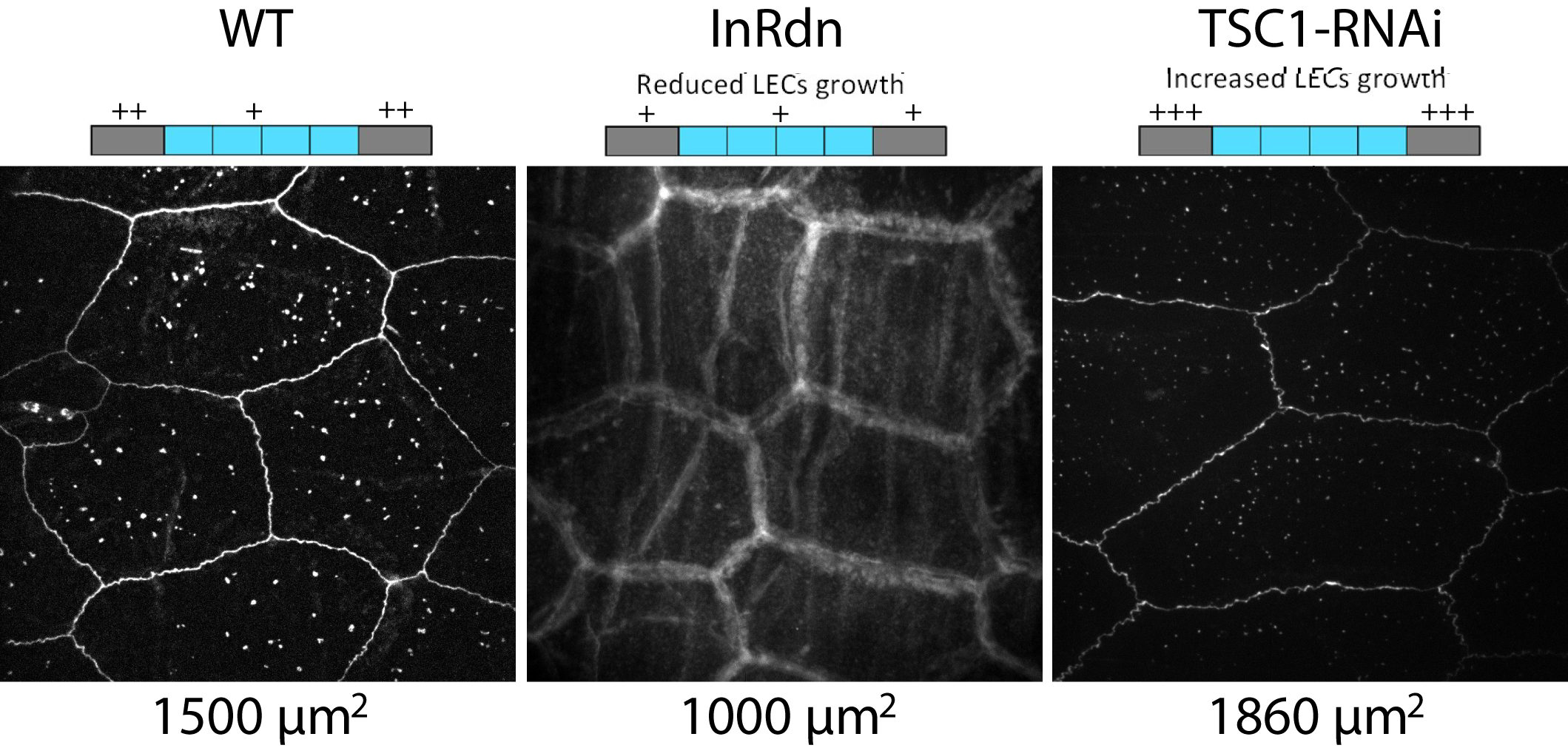

Supplement: S8 Fig — (A) Live imaging of the LECs of a WT wandering stage larva. (B) Live imaging of the LECs in which growth was reduced by impairing the insulin receptor pathway. (C) Live imaging of the LECs in which growth was increased by expressing TSC1-RNAi. The schemes above the images represent cell growth in larval epidermis. In the WT, LECs (in grey) and histoblasts (light blue) are growing at their physiological rate, with LECs faster than hb. This is indicated by 1 plus sign + for hb, and 2 (++) for LECs. In InR-DN larvae, LECs’ growth is slowed down, represented by a single + in the scheme. On the contrary, in TSC1-RNAi, LECs’ growth is increased, as represented by the 3 plus signs, +++. Scale bar = 10 μm. (TIF) [file pbio.3002662.s009.tif]

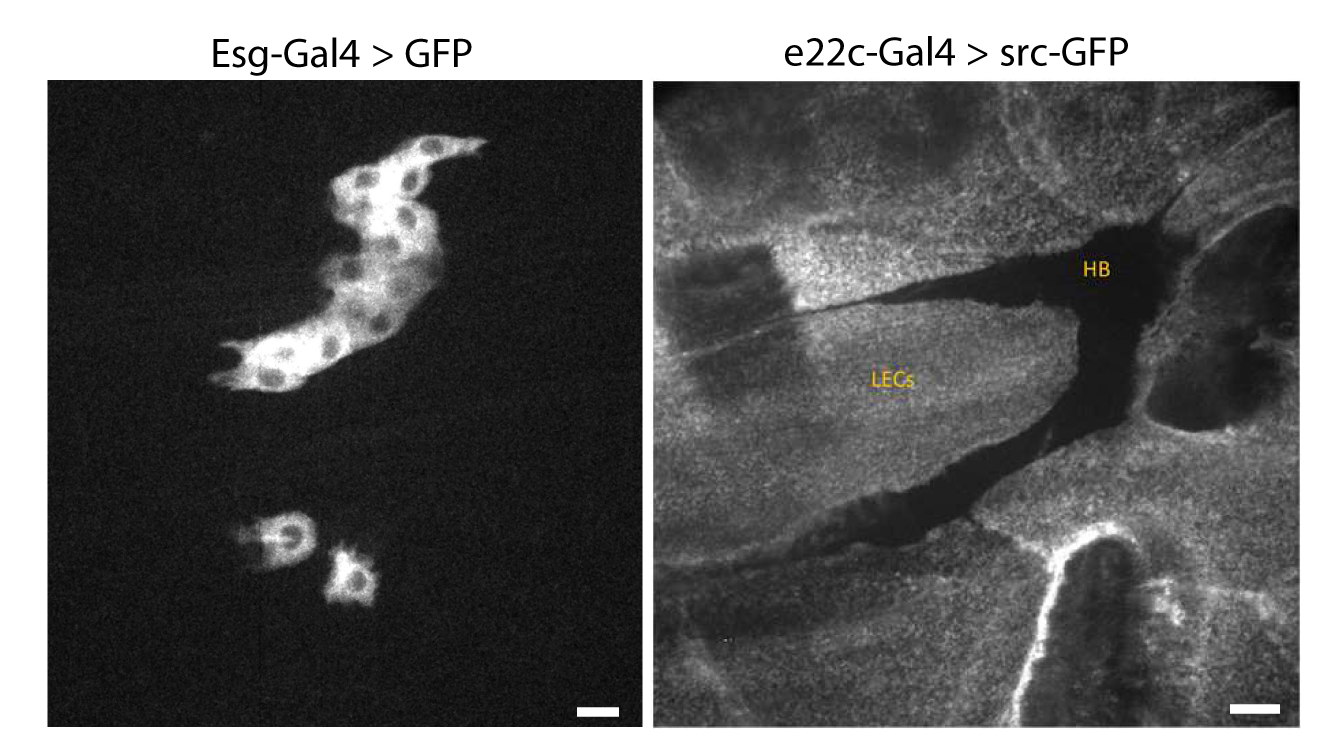

Supplement: S9 Fig — Left: Expression of cytosolic GFP under the Esg-Gal4 promoter. The image shows that the promoter of the escargot gene is specific to histoblasts and does not express in the surrounding LECs. The anterior and posterior dorsal nests are visible. Scale bar = 20 μm. Right: Expression of src:GFP under the e22c-Gal4 promoter. The image shows that the driver expresses in LECs, but not in histoblasts. The anterior nests is recognisable as a dark area. Scale bar = 20 μm. (TIF) [file pbio.3002662.s010.tif]
