# Supplementary material for: A mechanical transition from tension to buckling underlies the jigsaw puzzle shape morphogenesis of histoblasts in the Drosophila epidermis
Source: PLoS Biol. 2024 Jun 13;22(6):e3002662. doi: 10.1371/journal.pbio.3002662 (PMC11175506; doi:10.1371/journal.pbio.3002662)
Supplement: Attachment — Submitted filename: 124712_1_rebuttal_2635875_rs728q.pdf [file pbio.3002662.s014.pdf]

Reviewer #1 (Evidence, reproducibility and clarity (Required)):

The manuscript by Rigato and colleagues uses live imaging of Drosophila larvae to describe the transition of histoblasts from a configuration where junctions are straight to a configuration where junctions are folded. The emergence of folded junctions is concomitant with a decreasing apical area, an increasing cell height and decreasing junctional Myosin. Expression of Rab11-DN (reducing apical junctional trafficking) or Cdc25 (inducing proliferation) in histoblasts abolishes the emergence of folded junctions. Expression of InRdn (impairing insulin receptor pathway) in surrounding larval epidermal cells (LEC) is claimed to also reduce folding (but see comment below). Finally, laser ablation of folded junctions reveals little or no relaxation, indicating that these junctions are not under tension. The authors propose a model in which growth mediated cellular crowding generates mechanical constrain on histoblasts which, in conjunction with decreased junctional Myosin II, leads to the buckling of junctions.

o The authors convincingly show that histoblast junctions buckle/fold during late larval stages. They also convincingly show that junctional folding requires Rab11 and is counteracted by cell proliferation. The conclusion that folded junctions are not under tension is less well supported. Ablation of folded junctions results in little/no relaxation (Fig. 5), however, the positive control (late pupal histoblasts) did not show obvious relaxation either (in absence of quantification).

The reviewer highlights an important point and we apologize if our original manuscript was not clear on this point. We have added quantification of junctional relaxation after laser ablation (now Figure 4D). It shows that epidermal cells (derived from histoblasts) at pupal stage relax on ablation, whereas histoblasts at 3rd instar larvae barely relax. LECs show an intermediate behavior.

o The key conclusion that growth mediated crowding drives junctional folding is not well supported. The authors claim that InRdn expression in LECs leads to straight junctions of histoblast, but that is not obvious from Fig. 6F.These experiments will likely require several months.

Our discussion of the results was not well articulated in the original manuscript. We apologize for the lack of clarity. Our main conclusion, first stated in lines 121-126 of the new manuscript, is that junctional buckling is caused by the imbalance between the shrinkage of the apical domain of histoblasts and the continuous growth of their junctions. We have reorganized the manuscript to make it clearer. Figures 1 to 5 (Fig. 1-2: Phenomenology of histoblast shape; Fig. 3: Disruption of junctional growth; Fig. 4: Laser dissection; Fig. 5: Computer simulations) now focus exclusively on this hypothesis, which is also summarized in the cartoon of Fig. 8A (in the Discussion section). Up to this point, we do not address the question of why the apical domain of histoblasts shrinks, we only acknowledge it in a phenomenological way. However, this explains the presence of buckling. We have added a computer simulation of the mechanical process that supports our conclusion (see below).

In the last part of the paper (Figure 7, lines 235-266) we specifically address the size of the apical domain of histoblasts. We propose that increasing lateral pressure associated with growth on a limited pupal surface contributes to apical shrinkage of histoblasts (while they grow overall). To test this hypothesis, we modulate the growth of LECs. As in the original manuscript, we first express InR-DN in LECs (to reduce their growth). We have added a more precise quantification of the experiment and associated statistics. In addition, we have performed the opposite experiment where LECs’ growth is increased (via expression of TCS1-RNAi). Again, we provide a statistically sound quantification of the effect on cell size (histoblasts and LECs). Overall, we show that when LECs' growth is inhibited, histoblasts expand, and when LECs overgrow, histoblasts shrink apically. Note that histoblasts were not genetically manipulated in these experiments. Additionally, pressure effects impact mostly the

apical domain of the histoblasts as it's the only region of the cell in contact with LECs. These additional data and quantification support the hypothesis that mechanical stresses associated with growth affect the apical size of the histoblasts.

In the LECs undergrowth experiments, we observe a statistically significant reduction in the number of junctional folds (Fig. S5).

o Moreover, the authors provide a 'qualitative model' (Fig. 7). The paper would be strengthened by providing a model based simulation (best based on quantitative data derived from experiment (e.g. growth rates of histoblasts, LECs, etc.) of the transition of straight to folded junctions. This simulation would test whether the authors' hypothesized mechanism is feasible. This work will likely require several months.

The reviewer is absolutely right. A quantitative model was missing in the initial manuscript.

We collaborated with Richard Smith (John Innes Center, UK) and Adam Runions (University of Calgary) to simulate larval epidermal development. We could thus test how the formation of lobed junctions in histoblasts, could stem from a consequence of the imbalance between growth of junctions and shrinking of the apical area in histoblasts. As suggested by the reviewer, the model takes as input the experimental values for the decrease of apical area in histoblasts, and the increase of their perimeter. With this model, we were able to show that a compression of the nest, while junctions are growing, leads to buckled morphologies like the one we observe.

Elements and outcomes of the computer simulation are described in Fig 5, and discussed in the results section lines 179-209. Note that vertex models, often used to describe epithelial mechanics, are not adapted to pressure-driven mechanics (they are tension-driven). Our computational approach is thus original in the realms of epithelial morphogenesis.

o Statistical analysis to reveal significance between datasets is missing throughout the manuscript. The number of biological replicates (i.e. larvae) for each experiment are not provided. The authors should provide statistical analysis. Additional experiments will be required to further test the role of growth mediated crowding in junctional folding. For example, the authors should further attempt to modulate growth of LECs and/or test whether overgrowth of larvae to increase epidermal surface (e.g. lgl mutants) would influence junctional folding.

Following the reviewer's comment, we have added a thorough statistical analysis for all results presented in the manuscript. We have considerably expanded the morphometric description of figure 1, providing complete distributions of different quantities (Fig. 1H,I -see also Fig. 3E). All plots now use the box plot convention, which provides the min, max, 1st and 3rd quartile, and median), as well as all data points. We provide the important elements of the relevance of the statistical analysis in the figure panels through visual landmarks (*: p<0.05; **: p<0.01; ***: p<0.001; **: p<0.0001 and lower), provide all the p-values for all comparisons in the supplementary tables S1-S6, and added a section about the statistical analysis in the methods section.

As mentioned above, we now provide perturbative experiments for both undergrowth of LECs (InR-DN) and overgrowth (TCS1-RNAi). We show that when the growth of LECs is inhibited, histoblasts expand apically, and when LECs over-grow, histoblast shrink apically.

o Fig. 3/S3. The authors observe a correlation between junctional folding and a decrease in F-actin and Myosin on junctions. However, changes in Rok activity do not alter junctional folding. It is therefore unclear whether the decrease of F-actin and Myosin is a cause or consequence of junctional folding. The authors should tune down their conclusion that junctions 'soften' through a loss of cytoskeletal components.

The reviewer is right, the perturbative experiments were hard to interpret and did not bring any significant insight. From a purely mechanical standpoint, a change in junctional stiffness could impact buckling through a change in the critical load at which the instability proceeds. But thresholding effects in buckling are quite hard to reveal in general (it's a non-linear process), and the effects of a given cytoskeletal depletion on junction stiffness cannot be directly measured experimentally.

In the revised manuscript, we have very strongly tuned down our conclusions on the cytoskeleton, and simply mention that the observed cytosketal depletion could be a factor in promoting junctional softening, thus promoting buckling at a lower threshold. This is evoked in the revised discussion (lines 299-301). We are cautious in our affirmation ("a more systematic investigation of the link between the cytoskeleton and the stiffness of junctions must be established. ")

o Fig. 5. The laser ablation experiments require a quantitative analysis.

Quantification of laser ablation experiments has been added in the revised manuscript (added in Figure Panel 4D, and described lines 167-172). It shows that epidermal cells (derived from histoblasts) at pupal stage relax on ablation, whereas histoblasts at 3rd instar larvae barely relax. LECs show an intermediate behavior.

o Fig. 6. A control image (Cad;mKate without perturbation) is missing.

Following the reviewers comment, we have added WT controls for perturbative experiments (now Fig 3 and Fig 7).

o Fig. 7. The junctional buckling model is a hypothesis by the authors to explain their observation; it is not data and therefore should be removed from the 'results' section of the manuscript.

Following the reviewer's comment, we have encapsulated our results in a qualitative synthetic figure (Figure 8), which now belongs to the discussion. Note that we included a new figure that describes the computer simulation of the buckling process in the Results section (Fig 5).

Reviewer #1 (Significance (Required)):

The manuscript describes an interesting behavior of cells to change their junctional morphology from straight to folded during development. Previous work has mainly described epithelia as networks of cell junctions under tension. This work advances our understanding by providing evidence that epithelia can also be in a configuration that is not based on tension. The authors provide evidence that this cell configuration requires the absence of cell proliferation and trafficking of junctional material. However, beyond this, the mechanisms that drive epithelia into this configuration remain somewhat unclear. The manuscript would therefore likely target a specialist audience.

We thank the reviewer for the useful comments. As the reviewer acknowledges, we provide evidence of a novel biophysical regulation of cell morphogenesis. A fundamental point is that tension does not drive morphogenesis of histoblast, but rather compression. Until now, tension has been the main focus when it comes to describing epithelial cell morphogenesis. Compression was known to play a key role at very large scales (ex: brain convolution), or single biofilaments, but not in cell morphogenesis. For this reason, we believe that our case study should be of large interest, as it introduces the importance of compression in the definition of cell shape, thus linking different biological scales, and providing new information about epithelial morphogenesis.

In addition, we believe that our work can serve as a case study of tissues made up of heterogeneous cell populations, where spatial variations in growth can lead to compressive stress. Some analogies already exist with plant puzzle cells.

The revised manuscript is considerably improved from the original submission. Most importantly, we now provide a physical model of the onset of buckling using a computer simulation. The origin of buckling is quite simple: it results from an imbalance between the growth of junctions and the availability of space in the apical domain of the histoblast. The message is simple, but we think this is what makes the study interesting and potentially quite general. We are providing a mechanism for the morphogenetic process. This is a biophysical process, and we think that the description should remain at a general biophysical level, rather than trying to pinpoint a particular signaling pathway.

Reviewer #2 (Evidence, reproducibility and clarity (Required)):

In the manuscript "Compaction of Drosophila histoblasts in a crowded epidermis is driven by buckling of their apical junctions", Rigato *et al*. study the behavior of histoblasts during the third larval stage of Drosophila. Histoblasts are the progenitor cells of the adult abdominal epidermis. They are produced in the embryo and lie between the larval epithelial cells (LECs) in the larva before abdominal morphogenesis takes place during metamorphosis. The authors use this system to explore the role of forces during epithelial development.

The authors show that histoblasts undergo a dramatic change in morphology between 90 and 115 h AEL (after egg laying), with adherens junctions changing from a straight to a highly folded appearance. They also show that, during this time, histoblast volume is increasing. Furthermore, both junctional actin and junctional myosin II levels decrease over time. Interestingly, reduction of contractility (Rok-RNAi) and increase of contractility (overexpression of Rok constitutively active) in both histoblasts or LECs has no significant effect on histoblast behaviour.

The authors hypothesize that growth of histoblasts and LECs lead to a compression of the histoblasts, which causes the buckling of the histoblast junctions. To test this hypothesis, they perform three experiments and show that (1) over-expression of a dominant negative form of insulin receptor in LECs leads to a non-autonomous effect on histoblast cell size, (2) over-expression of a dominant negative Rab11 in histoblasts interferes with histoblast buckling, and (3) over-expression of String in histoblasts induces histoblast division and reduces histoblasts buckling.

Major comments

As mentioned in the significance box below, a major weakness of the manuscript is that the presented data does not sufficiently support the hypothesis that histoblasts are compressed by the LECs:

Firstly, the manuscript does not provide evidence that the LECs expand and compress the histoblasts. Such an analysis needs to be included.

We think that the perceived weakness stems from a lack of clarity in the statement of our working hypotheses in the initial manuscript. We have changed the structure of the manuscript in order to put forward in a clearer fashion our starting working hypothesis, which is that buckling proceeds through the imbalance between the continuous growth of junctions and the shrinking of the apical domain of histoblasts. Figs. 1-5 only focus on this working hypothesis, integrating the apical shrinkage as a phenomenological observation (Fig 1-2: phenomenology of histoblast shape, Fig 3: perturbation of junctional growth; Fig. 4: Laser dissection; Fig 5:

computer simulations). We then explore growth-induced pressure as a potential mechanism for apical shrinkage.

Regarding this latter point (growth-induced pressure):

- The growth of the two cell populations has been established in Madhavan (Madhavan & Schneideman, 1977). We refine their description by showing that while histoblasts do grow, they do so basolaterally.

- The apical domain of the histoblast-nest which is in contact (laterally) with LECs shrinks (Fig 1,2 in the revised manuscript). We do indeed propose that this shrinkage is linked to a mechanical load associated with the growth of cells on a limited surface (rather than an active contraction, for example). To prove this, we show in Fig 7 of the revised manuscript that a growth increase of LECs (TSC1 RNAi) shrinks the size of the nest and a growth decrease of LECs (InR-DN) expands the size of the nest. In these experiments, histoblasts were not targeted. Our interpretation is that the growth reduction (resp. increase) of LECs reduces (resp. increase) the growth-associated pressure in the epidermis.

- These results (including the new experiments of TSC1-RNAi) are presented in Fig. 7. There is now a separate section in the results specifically devoted to this mechanical tug of war ("A mechanical tug of war with LECs sets the apical size of histoblasts", line 235). We also provide comprehensive statistics on cell size in these perturbative experiments (Fig. 7D-F, tables S4,S5). They are also summarized in the discussion, Fig. 8B.

- Why did we alter the growth of LECs : (i) LECs contribute for a large majority of growth in the epidermis, because they occupy much of the epidermis surface. (ii) the non-autonomous effect is evidence that shrinkage of the apical domain doesn't necessarily rely on a specific signaling pathway within histoblast.

To conclude: we thank the reviewer for pointing to weaknesses in the initial manuscript. We do think that in revised manuscript addresses the point convincingly because :

i-The main novelty of the paper does not really rely on finding the exact origin of the histoblast apical shrinkage. If one accepts it phenomenologically (as the data demonstrates), buckling-induced morphogenesis is already a new, important result.

ii-We now provide statistically sound results of regulation of the apical domain of histoblast through LECs undergrowth and LECs overgrowth (which go in opposite directions). This is in strong support of the pressure-induced regulation.

Secondly, the authors present two pieces of evidence to support their hypothesis, which are not very convincing:

(1) In line 147ff, the authors imply that their experiments in Fig. S3D (Rok alterations in LECs) support a 'mechanical tug of war'. However, the authors point out that their analysis is not statistically significant, so their data does not actually support their hypothesis.

In line with our answer to the previous comment, we first clarify our main working hypothesis: buckling proceeds through the continuous growth of junctions in a shrinking apical domain of the histoblast. The main experimental evidence in favor of this hypothesis are the morphometric measurements, the perturbative experiments on junctions (Cad-RNAi, Rab11-DN), laser ablations, and the new computer simulation.

As discussed above, growth perturbations of LECs (overgrowth, undergrowth) support the mechanical tug-of-war hypothesis.

In the revised manuscript, we have very strongly tuned down our conclusions on the cytoskeleton, and simply mention that the observed cytosketal depletion could be a factor in promoting junctional softening, thus promoting buckling at a lower threshold. This is evoked in the revised discussion (line 301). We are cautious in our affirmation ("a more systematic investigation of the link between the cytoskeleton and the stiffness of junctions must be established. ")

(2) The authors show that over-expression of a dominant negative form of insulin receptor results in a non-autonomous increase in apical histoblast cell area. Here, I wonder to which extent the change in histoblast cell size might be due to a change in histoblast cell number per nest rather than a change in LEC pressure. Furthermore, they state that junctions are straighter, however, it would be good to see a statistical analysis here. Importantly, the conclusion of the experiment is not very convincing as it has not been shown that LECs are smaller, or grow slower, or exert less pressure on the histoblasts due to the performed genetic manipulation. So, the reason for the observed histoblast phenotype is unclear. This needs to be explored further.

The presentation of the InR-DN experiments of the initial manuscript lacked an in-depth analysis. In the revised manuscript, we have:

i- Quantified the size of LECs in WT and InR-DN which shows that InR-DN LECs are smaller than WT at the same age. The autonomous effect on LECs size is shown in Figs 7D, S6 (with statistically relevant quantifications) and is described in lines 247, 253-254 of the main text. We concomitantly demonstrate that, non-autonomously, the apical surface of histoblast is larger (Fig 7E). We also show that when LECs grow more (TSC1-RNAi), histoblast are smaller (again Fig 7D,E).

ii- Quantified the shape of histoblast junctions surrounded by InR-DN LECs, which shows that histoblast junctions are significantly straighter in the InR-DN LECs case than in WT. This is stated line 255, and presented Fig. S5, with details on statistics in table S6.

Note that in all growth perturbation experiments, the histoblast cell number remains the same. Not only were the histoblast not targeted by the genetic perturbation, but as histoblasts don't divide in the 2nd and third instars, the cell number remains the same in all our experiments (except when enforcing cell divisions through string over-expression). Furthermore, in all our experiments we never observed cell death, and the number of cells per nest remained tightly fixed (16+-3 WT, 15+-2 InRdn, 15+-3 TSC1 RNAi).

Thirdly, there are a few observations, which further challenge the author's hypothesis:

(1) Buckling appears to happen in the plane of the tissue, but should it not happen orthogonal to it, as shown in the scheme in Fig. 2J, if LEC compression was the cause of buckling?

The reviewer raises an interesting point. Indeed, we do not observe buckling in the orthogonal direction, as can be observed in figure 2. The only visible shape variation is the thickness of the cells, which increases.

In mechanical terms, buckling in the third dimension would be difficult as adherens junctions are sandwiched by the stiff apical extracellular matrix above, and septate junctions below. This laminar structure along the apico-basal axis is very asymmetric in mechanical terms. Thus, buckling is bound to proceed perpendicular to this axis. In line with the reviewer's remark, we added a comment in this discussion (line 304).

(2) In Fig. 1D, one can see buckling at interfaces between histoblasts and LECs - does this not suggest that histoblasts push against LECs (and that in that area LECs are not pushing against histoblasts)? I think this observation is very interesting and a more detailed analysis of this phenomenon at histoblast-LEC junctions could be included in the manuscript.

An interesting observation indeed. Note that even if it is likely that the pressure is higher in the nest, due to the higher growth of the surrounding LECs, LECs should still experience some degree of pressure. As a matter of fact, although there is a remnant relaxation when we ablate LECs, it's much smaller than that of the epidermis in the pupal stage for example. The interface junctions should then certainly feel an intermediate level of pressure. Furthermore, their heterogeneous nature should result in intermediate mechanical properties. It thus seems reasonable that they display an intermediate propensity to buckle. This is addressed in the discussion lines 296-303.

Based on the above point, there are various sections of the manuscript that need to be adjusted in my opinion, for example:

- Line 190ff. 'Combined, these perturbation experiments provide strong evidence that junctional buckling of the histoblasts is the result of an imbalance between the addition of junctional material in the histoblasts and mechanical constraints from the overcrowding of the epidermis.' - There is not sufficient evidence for this statement with respect to overcrowding. The presented data does however show convincingly that junctional remodeling is needed for buckling.

Following the reviewer's comment, the sentence has been removed.

Overall, in the revised manuscript, we are more careful about the interpretation of each experiment. We also have changed the structure of the manuscript. We introduce our working hypotheses step by step, more cautiously.

We first focus on the core of our working hypothesis, which has been rephrased into : "buckling proceeds through the imbalance between continuous growth of junctions on the one hand, and shrinking apical domain of the histoblast on the other hand (lines 121-126). At that point we do not explain why the apical domain shrinks, we just explore the consequences in terms of buckling. Owing to the statistically sound morphometry, perturbative experiments of junctions, ablation and computer simulations, we do think that we provide strong evidence in favor of this more clearly stated working hypothesis.

In the last part of the paper, we do explore a mechanism for the regulation of the size of the apical domain, which is the one mentioned above, linked to growth-induced pressure.

Line 201 ff. 'This experiments confirms that junctional buckling is a result of the combined overcrowding and absence of divisions.' - The presented experiments do not provide sufficient evidence for this statement. The presented data does however show convincingly that inducing cell division leads to less buckling. I wonder whether this result is counterintuitive with respect to the authors' compression hypothesis, as if increased compression from LECs would lead to buckling would then not also increased pushing by neighboring histoblast lead to buckling?

Following the reviewer's comment, and in line with the rephrased working hypothesis (see above), we have changed the sentence into: "This experiment further supports the model whereby buckling of the junctions proceeds because of their excessive lengthening while the apical area is constrained." (lines 153-154).

As we explain in the text (lines 144-146), in elasticity, the length of a rod is critical in defining its ability to buckle. In other words, it's much harder -for a given mechanical load and given rigidity- to buckle a short junction.

Line 235 ff. 'We investigated the formation of histoblast junctional folding and found that it is a non-autonomous transition originated by the competition for space of the two cell populations.' - This needs to be rephrased are there is not sufficient evidence for this hypothesis.

This sentence does not appear anymore in the reformulated discussion. The reformulated statement can be found at line 276 : "In our model, the phenomenological observation that the apical surface of the histoblast nest shrinks, while cell junctions grow, implies the compression-induced buckling of cell junctions and thus the formation of folds".

Further major comments

Line 56: More details about which histoblasts were imaged would help the reader understanding the experiments better.

- Which abdominal segment was imaged?

- The authors state that they have imaged the "dorsal posterior nest, which has the largest number of cells (15-17)" - is this correct? In later stages, the anterior dorsal nest is larger than the posterior dorsal nest.

The reviewer is correct, and we thank her/him for pointing out the mistake. The anterior dorsal nest is larger than the posterior dorsal, with 15-17 cells during the larval life. We imaged the dorsal anterior nest, which has a fix cell number between 15 and 17 cells. We have corrected the manuscript. (Line 60)

- Do the different abdominal segments have different histoblast numbers/ histoblast nest sizes?

- I think that it would be helpful to show the behavior of the ventral nest - the authors' hypothesis suggests that all histoblasts behave similarly and the buckling should be observed in all nests.

All abdominal segments have the same histoblast nests and cell number. All also display the same buckling process. We usually imaged abdominal segments 2-5, as beyond that,we found imaging less convenient due to curvature. The suggestion to show the behavior of all nests is a very good one. We thus have added Fig S2, which shows buckling in all nests of a given segment and the anterior dorsal nest of segments 1-6.

In the figures, the authors do not clearly present the statistics done. Here, giving the p-values in the graphs would be very helpful. There are instances where it is not sufficiently clear whether the authors present a significant finding or merely a non-significant tendency (e.g. in Fig. 6).

The reviewer is right. We have added a thorough statistical analysis for all results presented in the manuscript. We have considerably expanded the morphometric description of figure 1 providing complete distributions of different quantities (Fig 1H,I -see also Fig3E). All plots now use the box plot convention, which provides the min, max, 1st and 3rd quartile, and median). We provide some elements about the relevance of the statistical analysis in the figure planes through visual landmarks (*: $p<0.05$;**: $p<0.01$;***: $p<0.001$;**: $p<0.0001$ and lower), and provide

all the p-values for all comparisons in the supplementary tables S1-S6. We also have expanded the description of statistics in the methods section.

Fig. 4CD. I do not agree with the authors' interpretation that Sqh::GFP is lost from the junctions. The figure shows that levels are reduced. Also, in line 157, it might be better to use 'reduction of junctional myosin' rather than 'loss of junctional myosin'.

Following the reviewer's comment, we now use the terms :

" a gradual reduction of Myosin II enrichment at cell junctions" (line 220)

"a moderate loss of cytoskeletal components " (line 226)

With a few exceptions, the authors do not show the fluorescent marker used in their experiments to report where and how strong the Gal4 is expressed, which they use to drive their RNAi or dominant negative constructs. For example, in Fig. 5, they say that they have co-expressed a cytosolic GFP, but they do not show it. To have this kind of information would be very useful when interpreting the data.

Prior to perturbation experiments, we have tested the specificity of the primers esg-Gal4 and e22c-Gal4 at the stage of interest, by expressing a cytosolic UAS-GFP,. We added fig. S6 to show the expression pattern of e22c-Gal4 and esg-Gal4 used in the paper (resp. LECs driver and histoblasts driver).

Regarding the specific figure mentioned (now reorganized Fig 7 and Fig S5), we apologize for the lack of clarity that made the interpretation of the data unclear. We have corrected the captions of both figures. In InR-DN experiments, we used cadGFP to image junctions. These flies also carry a UAS-src:GFP. Hence, Fig. 7b shows two GFP reporters: cad:GFP, expressed ubiquitously, and src:GFP, which only labels LECs' membranes. The src:GFP generates a diffuse signal in LECs in Fig 7B. It is also clear from this image that there is no expression of src:GFP in histoblast (and thus no expression of InR-DN). The same holds for Fig S5B. Note that Fig 7C doesn't show the src:GFP signal because for these more recent experiments, we used cad:mkate as a junctional marker. Lastly, note that part of the diffuse cytosolic signal of Fig 3C is due the fact that Rab11-DN is fused with YFP which bleeds through in the GFP channel.

These occasional membrane or cytosolic signal in the same imaging channel than for cadherins were a constraint of the available genetic construction we had at the time of the experiments. But eventually they do provide an interesting piece of information : a measurement of the genetic pattern used for perturbation (again, in addition to fig S7).

Similarly, Rab11dn flies also carry a YFP. When crossed to esg-Gal4 cad:mKate flies, the generated larvae with GFP-labeled junctions, and a cytosolic YFP in histoblasts which is visible also in the GFP channel a blurry signal (fig. 3b).

In line 171 and in other places the authors talk about 'plastic remodeling'. It would be helpful if the authors could explain in more detail what they mean by this.

We call "plastic remodeling" a modification of the cell junction that leads to stress relaxation. This terminology comes from material science (wikipedia def: "the propensity of a solid material to undergo permanent deformation under load"). In polymer science, it usually implies a change in the structure of the polymer network. It naturally transfers to the cytoskeletal cortex: a change in the structure or composition of actomyosin must help relax the stress and consolidate the deformation. This is evoked in line 177 of the revised manuscript: "our data

suggest that some plastic remodeling must occur to stabilized buckled junctions and reduce the build-up of compressive forces. This could proceed through a change in the structure or composition of actomyosin in the junctions.”

Minor comments

In my opinion, the paragraph about the 'Qualitative model of junctional buckling' would be better placed in the discussion.

The qualitative model indeed has been moved to the discussion (Fig 8). There is now a computer simulation of the buckling process (Fig 5). In order to introduce the computer simulation we do give some “qualitative” elements in order to stress the difference between junctional buckling and simple buckling of an elastic rod (Fig 5A,B).

Fig. 6. Why was the insulin receptor experiment done in white pupa and not in wandering L3 larvae? This makes comparison of data more difficult.

The reason was that we used a gal80ts to control InR-DN in time -to induce the perturbation starting at L3. The changes in temperature (20 to 29 °C), and the deleterious physiological effect of the perturbation induced a great variability in developmental stages. Using white pupa ensured a better defined time window.

However, following the reviewers comment, the revised manuscript includes now measurements both at wandering and white pupal stages (Fig 7 and S5).

I wonder when are histoblasts stopping to show the buckling? I assume that it must be before the beginning of abdominal morphogenesis, as at the beginning of LEC replacement, the junctions are straight again?

Junctional buckling disappears at the very beginning of the pupal stage, when histoblasts go through fast division cycles that do not include the G2 phase (no growth) (Ninov, Chiarelli Martin-Blanco 2007). The fast division cycle retenses the junctions (as observed also in our ectopic expression of stg).

We briefly mention that histoblasts re-tense in early pupal life (line 326).

What is the morphology of histoblasts in late L1- and late L2-stage larvae? Potentially there might also be crowding during those stages?

We have imaged larvae at L2 and did not observe any buckling there.

Reviewer #2 (Significance (Required)):

The observations by Rigato et al. are very interesting. They present a novel model system that enables the study of the interaction of two epithelial cell types, which do not divide. The fact that the presented data suggests that neither histoblasts nor LECs are under tension, makes this an extremely interesting novel system to explore the forces involved. The presented results provide some interesting insights into histoblast biology. However, a major weakness of the manuscript is that the proposed hypothesis of histoblast compression by larval epithelial cells is not sufficiently supported by the results. So apart from the very

interesting observations, the manuscript lacks insight into the mechanistic basis of junctional buckling.

We thank the reviewer for stressing the novelty of our work. We think that with a better formulation of our working hypothesis, the added perturbative experiments, and the computer simulation, our manuscript now provides a strong insight into the biomechanical regulation of histoblasts, and the importance of buckling in cell morphogenesis.
