# Supplementary material for: A mechanical transition from tension to buckling underlies the jigsaw puzzle shape morphogenesis of histoblasts in the Drosophila epidermis
Source: PLoS Biol. 2024 Jun 13;22(6):e3002662. doi: 10.1371/journal.pbio.3002662 (PMC11175506; doi:10.1371/journal.pbio.3002662)

ANSWERs TO REVIEWERS COMMENTS

Reviewers' comments
Rev. 1:
The authors have satisfactorily addressed most of my comments and the manuscript is much improved. I have some remaining comments:
Fig. 3B. The authors claim that cell junctions are straighter when E-cadherin is depleted. However, the cell junctions (labeled by E-cad::mKate) are difficult to see. Thus, it is difficult to assess whether Cad-RNAi results in straighter junctions (the authors are also unable to segment the images for shape quantification (line 136)). The authors should use a different marker for cell junctions (or plasma membrane) which is not affected by E-cadherin depletion.
Following the reviewers' comments, we performed cad RNAi experiments with src:GFP – a membrane marker that we already used in Fig. 2G on the WT. The effect of cad-RNAi is stronger in this genotype (esg-Gal4 UAS-src:GFP UAS Cad-RNAi). From all the wandering stage larvae that we imaged, we observed no junction folds/lobulations. In the experiment of the previous manuscript, we probably biased the RNAi phenotypes by searching for larvae where cadherin was still visible (i.e. which presented a weaker depletion). We added the images of WT and CadRNAi wandering stage nests in Fig. 3D,E. In both cases, one can see the apical shape, with strong folds in the WT and no folds in cad-RNAi. Note that in both cases, one can see the rounded basal side, which proceeds at the wandering stage. We added statistics in Fig. 3G-I.

Fig. 3E. The authors should state in the legend what the color code refers to.
The color code was initially in the caption. Indeed, the reviewer is right that it is also needed in the figure panel. We thus added it.

Lines 195-208. The authors refer to Fig. 8E-H; Fig. 5 would probably be correct.
We thank the reviewer for pointing out the mistake, which has been corrected.

Fig. 7C. The authors should state which marker (cad...) they have used.
We added this information in the figure caption, cad::mkate was used to mark cell junctions. We also have indicated all genotypes of the study in table 2.

Fig. 7C. The authors show that increased LEC growth (TSC-RNAi) results in reduced histoblast area. To further test their hypothesis, the authors should determine whether in this situation also the buckling of cell junctions (circularity) is affected (like the authors do for the reduced LEC growth (Fig. S5)).
This is a challenging question. There is indeed an effect on lobule shape, narrower and more packed, as visible in Fig 7. This effect verified by the quantification of the junction straightness (defined by the ratio of end-to-end Euclidean distance over the curvilinear distance along the junctions), which decreases from 0.67 (WT, median value) to 0.57 (tsc1-RNAi, $p = 0.004$).
However, we do not measure a significant change in the circularity value. We believe this is due to two main reasons.
First, the calculation of circularity depends on the precise measurements of cell area and perimeter. But in e22c>tsc1-RNAi TSC1-RNAi larvae, the nests are so shrunk that our segmentation fails, and individual cells are not detected correctly. Quite frequently, the opposite cell membranes even come into contact, transforming one convolved cell into several less convolved rounder cells (see figure below). Note that the straightness is less sensitive to this because a full segmentation of the cell is not required (segmented junctions or pieces of junctions are enough). We have added the statistics on straightness index lines 300.
See below how one orange cell with low circularity can be transformed by segmentation error into 3 cells, 2 with high circularity and one with intermediate. This messes up the statistics. We haven't found an easy way (area cutoff) to remove these artifacts.

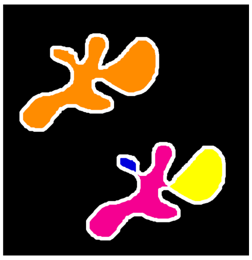

	Area (px)	Perimeter (px)	Circularity
1	34341	1534.219	0.183
2	19006	949.235	0.265
3	12657	458.357	0.757
4	1058	137.983	0.698

Second, we believe that there is also a non-autonomous effect on the growth of histoblasts' junctions. In fact, we observed decreased cell perimeter, that cannot be explained only by the artifact mentioned above. This could be expected, as a compressed cell is likely to yield contact inhibition-type feedback, complex to analyze.
Nevertheless, the experiment does provide useful information. In particular, it adds evidence of the tug-of-war mechanism that controls the apical nest size and, consequently, the junction shape.

****

Rev. 2:
As I stated in my first review, the observation of histoblast junctional buckling is very interesting. Also, the authors' hypothesis, presented at the end of the discussion, that histoblasts buckle to ensure that they are not accidentally pushed out of the tissue/die during larval growth is intriguing.

In this second revision, we present new experiments which strengthen our mechanical description of histoblast. We confirm that indeed histoblasts are in a mechanical state different from the one of embryonic tissues, for example. We think the work will interest a wide audience working on tissue mechanics in a developmental context.

Overall, the authors have sought to address all my comments and have improved the manuscript. As part of the revision, the authors have adapted their line of argument. Based on their data, the authors formulate the hypothesis that junctional lengthening combined with a reduction in histoblast area leads to junctional buckling. For some reasons histoblasts can't withstand this pressure like LECs.
Indeed, as growth proceeds on the epidermis (LECs and histoblast), pressure builds up. This pressure varies smoothly over the surface of the epidermis and both histoblasts and LECs probably experience it. As we argue in the discussion lines 342-348, there are several possible factors that make histoblasts more compliant, and more prone to buckling of their junctions than LECs.

Although the revision has improved the manuscript, two main points remain, which in my opinion need to be addressed further before the manuscript can be published in PLoS Biology.
We thank the reviewer for stressing some potential misunderstandings regarding the mechanics of histoblasts. This pushed us to clarify our statements and make important additional experiments. Some of them (optical tweezers on living larva) were a real technical challenge. But they helped us to strengthen our point. See below.

1) The manuscript does not sufficiently explore the mechanism behind the behavior of the buckling junctions.
See below for our specific answer on histoblast junction mechanics: we now provide evidence that the mechanics of junctions are dominated by elasticity. As they grow in a constrained domain, they buckle.

How are the forces created that lead to junctional lengthening, and what stabilizes the buckled junctions?
Similar to other epithelial systems, a major driving force is the accumulation of junctional adhesive proteins in these growing cells. Our experiments show that altering trafficking of the endocytic pool (rab11-DN) or reducing the total production of cadherins (Cad-RNAi) results in smaller junctions and no buckling. This is a crucial point, and our previous manuscript did not thoroughly explore the cadRNAi experiment. Consequently, in this revised manuscript, we present new cad RNAi experiments in which we observe the apical side of histoblasts with a membrane marker. The phenotype is impressive, and we observed no lobulations in all the wandering stage larvae that we analyzed. An image has been added to Fig. 3D,E, and the quantifications are included in Fig. 4G-I.
In growing dividing cells, junctional material increases, but junctional growth is balanced by cell divisions, limiting the growth of an individual junction to the cell cycle duration. However, when we consider the integral of junctional length at the cell population level, it does show growth. In these other systems, no buckling occurs because 1) junctions are kept short by continuous cell divisions, as discussed in the cdc25 experiments (lines 164: 'Would the histoblasts divide like, for example, cells of the imaginal discs, the deep junctional lobules would not occur'); 2) these systems are usually not under compression.
'What stabilizes the buckled junctions?' : In an inert elastic system, one would expect the buckled shape to persist as long as compressive stress is maintained. However, in cells, quasi-static processes associated with the renewal of junctional components and growth come into play. This is what we address in the ablation experiments, which we refer to as the plastic process (see below).

Laser ablation experiments show that there is hardly any relaxation when junctions of buckled histoblasts are cut, which the authors interpret as a 'plastic process [that] dissipates the compressive stress'. What is this process?
We observe no relaxation upon ablation, thus the stress that generated the deformation has dissipated. Plastic processes are non-reversible changes of shape in response to applied forces. Because junctions undergo important

deformations, it is plausible that the dissipative process at play in junctions is of plastic nature. However, we have no formal proof and following the reviewer's comment, we have used predominantly "a dissipative process" in the revised manuscript. We have added (line 230) a comment saying that as of now, we do not know if the stress dissipation is triggered by deformation (plasticity), or if it is time that matters (viscosity). Note that we demonstrate with new experiments (see below) that on a short time scale, histoblasts are elastic (no dissipation). Thus, our current mechanical interpretation of late larval histoblasts is that of an elastic structure, which dissipates only at a very long time/ deformation. Most importantly, in our work, we show in the simulation that this is compatible with the existence of buckling.
Regarding the molecular nature of the dissipative process, we speculate in the simulation section (line 229) and in the myosin II section (line 273) that it could stem from reorganization of the cytoskeleton.

In addition, the model includes 'an elastic fabric impeding the rapid displacement of the boundary'. What is this 'elastic fabric'?
see below for a detailed answer. Note that we have decided to change the terminology for the more canonical expression (in mechanics terminology) "elastic foundation".

Do these observations suggest that the junctions, although they are strongly bent, are very stiff/rigid? Would this depend on a rigid cortical actomyosin network?
We do think indeed that they are elastic structures (in the sense that they are not as dissipative as embryonic junctions). This is a central point that we did not explained clearly enough in the previous manuscript- and we thank the reviewer for pointing this out. As suggested by the reviewer (below), we have added analysis from live imaging to show that the tissue is really static compared -for example- to embryonic tissues. This corresponds to Fig S3. This figure is first mentioned in lines 73-79 in the text. We then refer to it again line 189. In this revised manuscript, we investigate more thoroughly the mechanics of histoblasts (paragraph starting line 188) by adding a critical experiment : optical tweezers that show that when you stimulate a point on a junction, the entire region moves in synchrony. The overall structure is elastically interconnected. This corresponds to Fig 4E-H, Movie S1 and lines 190-207 in the text. By comparison, the same experiment in embryos does not display such an interconnection : the mechanical oscillation does not propagate to neighboring regions (this has been reported previously in Clement et al. cited in our manuscript and we reiterate the measurement by sake of completion in fig S5, and Movie S2). Moreover, we show in the tweezer experiment that different junctions seem to be interconnected through the cell medium (cytoplasm or apical cortex). This elastic connection serves the role of an "elastic foundation" (a classic mechanics terminology) that prevents large, low-mode deflection of the beam. In the previous manuscript we had used the term "elastic fabric" thinking this could be more intuitive when talking about a tissue. Clearly, it was a mistake and we now stick to "elastic foundation". While indeed this very likely depends on an elastic cortex, we stress that the whole apical domain of histoblast may become elastic through its connection to the stiff apical extracellular matrix (mentioned line 343).
Note that the junctions are elastic on short time scales, but on longer times scales they adapt to their bent morphology (what we call the dissipative process). As we mention in the discussion (line 339), this is reasonable that on the time scale at which growth operates, there is some renewal of junctional components that could help release mechanical stress.
Overall, through the added experiments, and the reformulation of our interpretation of experiments, we think the revised manuscript is clearer on the description of histoblast mechanics – making the results stronger and all the more original.

Here, the actin and myosin data seem counterintuitive. This highlights that the role of the described changes in actomyosin is not sufficiently clear.
First, and as we mentioned in the previous version of the manuscript, we have toned down our statements with respect to the cytoskeleton since our first version of the manuscript. But we want to mention that 1) while we state that junctions in histoblast are elastic rather than dissipative (except at very long times scales), a reduction in stiffness can still happen, which would promote buckling through a reduction of the critical buckling load, rather than impede. 2) An enrichment in actomyosin in the apical medial medium could increase the stiffness of this region and promote elastic coupling of junction to the elastic foundation.
These elements are discussed in lines 264-270 in the results section and 348-349 in the discussion.

To explore the properties of the buckling junctions in more detail, live imaging would be useful. From the authors' data, one would expect that the buckled junctions are very stable and do not change much over time (in contrast to dynamic changes in the buckling morphology, which one would expect if one would deform a flexible, already buckled rod).

Such live imaging would also help to illustrate the statement made in line 74: 'No fluctuations of the cell junctions are observed in a period of minutes.'.

As detailed above, following the reviewer's comments, we have performed live imaging. The junctions are indeed static -- or rather quasi-static as there are still some slow dynamics (building-up of lobules as the junctions grow). We also have performed optical tweezers experiments which bring new assessment of our hypothesis. It stands that when mechanically stimulated at one point, the whole surrounding moves in a quite coherent fashion (see movie S1). We thus fully agree with the reviewer in their above statement. The corresponding changes in the manuscript text and Fig 4 have been detailed above.

2) The manuscript still does not provide sufficient support for the hypothesis that pressure from the LECs is causing histoblast buckling.

One experiment that could expand on the InR-DN and TSC1-RNAi experiments would be to ablate one LEC neighboring the histoblast nest to release tension and ask if and how junctional morphology of the histoblasts changes.

While this is a very interesting suggestion, we think that such an experiment is likely to generate active processes of wound healing and compensatory growth that would make it hard to interpret.

For the InR-DN and TSC1-RNAi experiments, it would be helpful, if the authors explained in more detail which LECs were assessed for their cell area (all of them, those neighboring the histoblasts?).

We measured the area of LECs within 3 cell diameters of histoblast nests. We measured LECs area on the same larva that were used for histoblast measurements -preventing variability associated with fitness and precise developmental stage. Owing to the fact that the same buckling occurs in dorsal, ventral, anterior, posterior nests of all segments, we assume that there is no regionalization of LECs growth either. Most importantly, for the perturbative experiments, e22c-Gal4, which is an epidermis driver used in many studies, drives expression in all LECs, in an unregionalized fashion (see below, last question).

If all LECs of the abdomen got bigger or smaller, would this not have strong effects on epithelial morphology, as a large area is either created or lost (or are the larvae smaller/larger)? Are LEC numbers in one segment similar in InR-DN, TSC1-RNAi and control experiments?

The change in LECs is not immense. This is at least partially buffered by compression of histoblast nests. Note that because LECs cover a large portion of the epidermis, a small change in LECs can mean a big change for histoblasts. Note that there is no cell divisions in LECs in the developmental stages that we analyse. Thus there is no change in cell number in these experiments.

Also, it would be useful to know not just the cell area of individual histoblasts, but also the overall area of the nests in these experiments. This would indicate the area available for the histoblasts and further highlight the change in available space for the histoblasts. (This would also be interesting for the wild-type analysis in Fig. 1).

Following the reviewer's comment, we also include the change in area at the nest scale, lines 303-307, where it is indeed quite relevant.

Also, does junctional tension change in inR-DN and TSC1-RNAi LECs?

Could a detailed study of the morphology of LECs and histoblasts by live imaging at the start of buckling help to explore whether LEC pushing is involved? For instance, does buckling begin in certain areas of the nest, which maybe correlate with specific LECs getting larger? Also, the authors discuss that buckling begins later at the LEC/histoblast interface - is this because of more rigid junctional tension or because LECs actively push?

We do not say in this or the previous version of the manuscript that LECs are pushing.

Throughout the manuscript, we talk about pressure build up, as cells grow, instead of "LECs pushing". Our view is not of LECs performing an active process against histoblast at the LECs-histoblast interface. Although an interesting idea, we haven't seen anything like this. Rather, as growth proceeds on a limited surface, pressure builds up --ie compression. In a way, histoblast could contribute as well to this pressure build up. But they occupy a much smaller surface, and grow slower.

To prevent any possible misunderstanding, we strived to use the proper terms in the revised manuscript. Straight from the first introduction of the buckling hypothesis, we make it clear that it is a matter of a balance between growth of junctions and growth/shrinkage of the apical domain of histoblast (line 127). We repeat this at several other instances -for example when we introduce simulations (line 209-213), where we stress that potentially, buckling could proceed with an apical domain that is stationary or even grows - if this growth is slow enough that it does not balance the fast growth of junctions.

In response to the reviewer's comment on the location of buckling, there is something very interesting that we're exploring currently --not about position but orientation. There is an asymmetry in the change in nest shape: it shrinks more along the antero-posterior axis. and on average the junctions that buckle first are oriented along the antero-posterior axis. This is something we're currently exploring, but remains for a future study.

Lastly, on the LECs/histoblast interface : We do think indeed that these junctions have intermediate mechanical properties which make them buckle less prominently as mentioned in the discussion (lines 346-349).

It would also be interesting to explore the changes in LECs area in the whole hemisegment. Are all LECs growing? Is the change in LEC shapes consistent with a potential increase in pressure on histoblast nests?
We refer to madhavan et al 1977 (ref 27) for an analysis of the growth in the epidermis, which included regional differences between LECs and histoblasts. If LECs in one region were growing significantly more we would not observe this following pattern of cell (nuclei) distribution which is quite regular.

from Madhavan 1977:

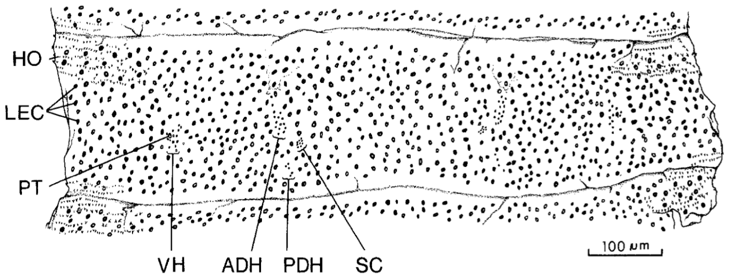

Fig. 14. A semidiagrammatic camera lucida drawing of a whole mount of the epidermis of the fourth abdominal segment of a 44 h-old larva (same segment shown in Fig. 13). The whole mount was prepared after dissecting the larva mid-ventrally and shows the distribution of the paired anterior dorsal (*ADH*), posterior dorsal (*PDH*), and ventral (*VH*) histoblast nests. *HO* hooks; *LEC* larval epidermal cell; *SC* small cells; *PT* polytene cell

From all our own observations we observe similar regular cell lattices of LECs at later stages as well. If there was a small degree of regionalization of LECs growth, this would not affect our conclusion. The overall tissue would still experience an overcrowding and pressure increase (note that pressure is smoothly varying in space). This could affect, for example, the exact time at which different histoblast nests go through the buckling transition. But note then that we observe the same buckling process in dorsal, ventral, anterior, posterior nests.
Eventually, the strongest point in favor of the growth induced pressure is the perturbative experiments of LECs growth, which affect the size of the histoblast nests (with a perturbation that affects all LECs, see below).

Further points:
I wonder whether Fig. 6 should come before Fig. 5, because the authors talk about actomyosin at the junctions when they discuss Fig. 4 (line 177 'This could proceed through a change in the structure or composition of actomyosin in the junctions.').
We hesitated quite a lot about moving the myosin figure. Indeed the reviewer's suggestion is interesting. We finally kept the myosin experiments in Fig 6 because this figure is here in support of our working hypothesis developed through a mechanical analysis rather than a strong new statement in itself. The figure is here to show that it's compatible with our buckling model.

The authors could show a quantification of cell shapes for the experiments in larvae presented in Fig. 7. They only show the quantification for pupae (in Fig. S5).
In the dInR experiment we had to integrate the perturbation for long enough for a significant effect to be measured. The onset of perturbation had been limited to 3rd instar (using gal80ts) because before that e22c also drives in histoblasts.

Also, a reference to Fig. S5 is missing in the main text.
We have added a reference to this figure, now S7.

The authors should include the number of histoblasts for each experiment to make clear that the phenotypes are not due to a reduced cell number. For some experiments, the authors state these data in the response to the reviewers, but the data are missing in the manuscript.
This information is now present in each figure caption.

It would also be useful to know how many larvae/pupae were analyzed for each experiment. Currently, only the n-numbers for the number of analyzed cells are given in the manuscript.
We have added this information in the Statistical analysis section of the manuscript.

In Fig. S4, the n-numbers are highly variable. In particular, there is only a single data point for a circularity of 0.7 - this n-number needs to be increased.

The numbers are highly variable because this is an analysis done *a posteriori.* We agree with the reviewer, one single data point is not representative. We removed this data point, which is not significant, but won't be able to increase the statistics of this one point.

Paragraph starting at line 196: figure numbering is incorrect here.

We have corrected the figure references.

Line 349: a figure reference is missing.

Fig. S3 legend: There is an incomplete sentence - 'E Ratio of basal/apical area for different circularity values, obtained from .'.

Now Fig. S4 "E Ratio of basal/apical area for different circularity values, obtained from the masks of the z-projections as shown in C and D, i.e. z2/z1 ".

Fig. S5: The yellow arrows could be explained.

The yellow arrows are a visual help to highlight the change of LECs' size in TSC1-RNAi and InR-DN larvae, compared to the wild type. We added a sentence to explain this.

Fig. S6: The schemes above the images could be explained in more detail - they are not fully clear without consulting the other figures that have these schemes.

We added an explanation of these schemes in the caption.

"The schemes above the images represent cell growth in larval epidermis. In the WT, LECs (in gray) and histoblasts (light blue) are growing at their physiological rate, with LECs faster than hb. This is indicated by one plus sign + for hb, and two (++) for LECs. In InR-DN larvae, LECs's growth is slowed down, represented by a single + in the scheme. On the contrary, in TSC1-RNAi, LECs's growth is increased, as represented by the three plus signs, +++."

Fig. S7, right image: This is interesting. It would be useful to see a lower-magnification image of the whole segment, because, in pupae, en.Gal4 only drives expression in the posterior compartment (in both LECs and histoblasts). Also, which nests are shown? In pupae, I would expect en.Gal4 expression in the dorsal posterior histoblast nest.

(Now Fig S9) As specified in the beginning of the results section (lines 59-60), we image the anterior dorsal nest, because of the larger cell number. As the images of Fig S9 show, we did not detect any expression of src::GFP in the histoblasts at the late larval and pupal stages. Furthermore, this epidermis driver, e22c-Gal4, expresses in all LECs of the epidermis at larval stage, irrespective of the compartment. It is used as a standard epidermis driver by many labs. See below the low magnification image of 3 segments in an e22c-Gal4 UAS-src:GFP genotype. Although not sectioned (hence the background haze), one can see that cell membranes are labeled throughout the segments.

Original *Boosted gains to see cell outlines (src:GFP)*

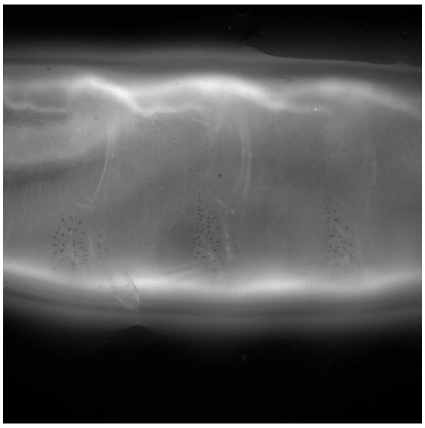

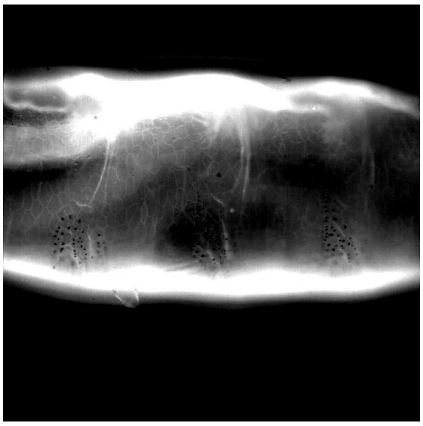

Supplement: Attachment — Submitted filename: Rigato_answers.pdf [file pbio.3002662.s016.pdf]
